# Hop Extract: An Efficacious Antimicrobial and Anti-biofilm Agent Against Multidrug-Resistant Staphylococci Strains and *Cutibacterium acnes*

**DOI:** 10.3389/fmicb.2020.01852

**Published:** 2020-08-13

**Authors:** Silvia Di Lodovico, Luigi Menghini, Claudio Ferrante, Erica Recchia, Juliana Castro-Amorim, Paula Gameiro, Luigina Cellini, Lucinda J. Bessa

**Affiliations:** ^1^Dipartimento di Farmacia, Università degli Studi “Gabriele d’Annunzio” Chieti-Pescara, Chieti, Italy; ^2^LAQV/REQUIMTE, Departamento de Química e Bioquímica, Faculdade de Ciências, Universidade do Porto, Porto, Portugal

**Keywords:** hop extract, *Staphylococcus* spp. and *Cutibacterium acnes*, antibacterial activity, antibiofilm activity, antimicrobial resistance, biocompatibility

## Abstract

Bacteria belonging to *Staphylococcus* genus, in particular methicillin-resistant *Staphylococcus aureus* and multidrug-resistant *Staphylococcus epidermidis*, together with *Cutibacterium acnes* are the main strains involved in skin disease. The increase in multidrug-resistant bacteria has revived attention on natural compounds as alternative agents for the treatment management. Among these, hop extract, a hydroalcoholic solution obtained from experimental crops of *Humulus lupulus* L. variety *cascade* (hop), displays diverse biological properties including an antimicrobial one. The aim of this study was to evaluate the antimicrobial activity and the capacity to inhibit the biofilm formation of a characterized hop extract against *S. aureus* and *S. epidermidis* multidrug-resistant strains and against a *C. acnes* strain. The hop extract was characterized by (i) phytochemical analysis through a reversed-phase high-performance liquid chromatography (HPLC)–fluorimetric method, (ii) biocompatibility test with *Artemia salina* L., (iii) cytotoxicity against two cell lines, (iv) docking analysis, and (v) antimicrobial and antibiofilm activities by detection of zones inhibition, minimal inhibitory concentrations (MICs), biomass quantification, and cell viability. The hop extract was biocompatible and non-cytotoxic at all tested concentrations. HPLC analysis revealed significant levels of gallic acid, resveratrol, and rutin. This last compound was the most representative displaying a high affinity against PBP2a and KAS III (Ki values in the submicromolar range). The characterized hop extract showed a good antimicrobial action with MICs ranging from 1 to 16 μg/mL and was able to inhibit the biofilm formation of all tested strains, except for two *S. aureus* strains. The biofilm formed in presence of the hop extract was significantly reduced in most cases, even when present at a concentration of 1/4 MIC. The live/dead images showed a remarkable inhibition in the biofilm formation by hop extract with a weak killing action. Overall, the tested hop extract is a good candidate to further explore for its use in the prevention of infection particularly, by multidrug-resistant Gram-positive pathogens.

## Introduction

Nowadays, the increase in multidrug-resistant (MDR) bacteria represents an important global emergency that encourages the search for new strategies to overcome this complex phenomenon that led to the failure of traditional antibiotic treatments. Diverse studies show that infections by MDR strains are one of the principal causes of morbidity and mortality (Levin-Reisman et al., 2017; Oliveira et al., 2018). This phenomenon involves pathogens isolated from different sites. The higher number of MDR Gram-positive microorganisms coming from skin infections with a high percentage of methicillin-resistant *Staphylococcus aureus* (MRSA) (Lu et al., 2018) is significant.

Staphylococci are the main strains colonizing the resident human microbiota, and in particular, *Staphylococcus epidermidis* and other coagulase-negative staphylococci are deemed major skin commensal bacteria (Prescott et al., 2017). Coagulase-positive *S. aureus* is not considered to be part of the natural skin microbiota, and it is able to colonize different sites causing infections such as bacteremia, pneumonia, endocarditis, and toxic shock syndrome (Becker et al., 2014; Peacock and Paterson, 2015). If the cutaneous and mucosal barriers are compromised, and the microbiota is unbalanced, *S. aureus* can gain access to underlying tissues, causing opportunistic infections (Grice et al., 2008; Coates et al., 2014; Williams and Gallo, 2015; Lee et al., 2018).

The emergence of MDR *S. aureus* and *S. epidermidis* strains has led to a high number of infections toward which of the treatment and control has been failing. In particular, the increase in MRSA and vancomycin-resistant *S. aureus* strains is a challenge in the actual clinical practice (Oliveira et al., 2018). MRSA are microorganisms responsible for most skin and hospital-acquired infections, associated with a high level of mortality (Bocquet et al., 2019). The methicillin resistance is due to the acquisition of gene encoding for a penicillin-binding protein 2a (PBP2a), reducing the β-lactam action (Peacock and Paterson, 2015). Furthermore, both *S. aureus* and *S. epidermidis* have the capability to form biofilms, which are also implicated in the pathogenesis of numerous bacterial infections caused by those bacterial species (Weber et al., 2019). The presence of biofilms reduces the effectiveness of the antimicrobial agents, increasing their tolerance level.

*Staphylococcus aureus* is often identified along with *S. epidermidis* and *Cutibacterium acnes* as the main acne-causing bacterium (Yamaguchi et al., 2009; Sinha et al., 2014).

*Cutibacterium acnes* is an important skin commensal that it is also able to become an opportunistic pathogen, causing invasive infection of the skin, soft tissue, cardiovascular system, or deep-organ tissues (Achermann et al., 2014). This bacterium produces β-ketoacyl-ACP synthase III (KAS III) involved in fatty acid synthesis, lipases, proteases, and hyaluronidases injuring the tissue lining of the pilosebaceous unit and encodes for immunogenic factors with adherent properties and porphyrins (Coenye et al., 2007; Isard et al., 2011; Platsidaki and Dessinioti, 2018).

The antibiotic resistance/tolerance phenomenon has revived the attention on plant extracts as promising agents with antibacterial and antibiofilm activities.

*Humulus lupulus* (hop) belongs to the Cannabaceae family and is used in the brewing industry for its bitter and aromatic properties. The plant presents secretory structures on the epidermal surface of stem and leaves, but only the female cones are of economic relevance for the greater presence of secretory glands, which are easily separated from dry plant material. The powder material represented by only secretory glands results the most expensive and is defined as lupolin. In such glands, terpenoid metabolites are synthetized and stored, such as bitter acids, terpenophenolics, monoterpenoids, and sesquiterpenoids. Those compounds are usually responsible for typical flavors, which therefore can be obtained using isolated glands (lupolin) and whole cones. Hop extract and relative compounds such as polyphenols and acylphloroglucides have also been used in the cosmetic and pharmaceutical industries because of their antimicrobial and antiviral effects. In ancient times, hops were used against leprosy, bad smell of feet, liver diseases, constipation, sleeping disorders, and for blood purification. Moreover, alcoholic extracts of hops have been used in ayurvedic medicine for treating pulmonary tuberculosis and acute bacterial dysentery due to their strong spasmolytic effect on smooth muscle (Blumenthal et al., 2000; Olsovska et al., 2016).

Previous studies (Gerhäuser, 2005; Olsovska et al., 2016; Weber et al., 2019) showed that xanthohumol and lupulones, prevalent hop active compounds, inhibit *C. acnes*, *S. epidermidis*, *S. aureus*, and *Streptococcus pyogenes* growth.

On that basis, the goal of this study was to characterize a hydroalcoholic solution of hop extract and evaluate its antibacterial action and its ability to inhibit biofilm formation against clinical MDR isolates of *S. aureus*, *S. epidermidis*, and also against a susceptible strain of *C. acnes.*

## Materials and Methods

### Plant Material and Phytochemical Analysis

Hop extract was kindly furnished by Bioinvest S.r.l. It consisted of a pale yellow hydroalcoholic solution obtained from experimental crop of *H. lupulus* L. variety *cascade* grown in Abruzzo (Italy). After manual collection and selection of female cones, the plant material was dried in ventilated oven (40°C) until achievement of a constant weight. Dry plant material, with water content lower than 10%, was extracted in 60% ethanol in 1/20 plant material/solvent ratio (wt/wt). Temperature and extraction process were optimized for extract recovery, stability, and organoleptic note, and the resulting extract is characterized by typical phloroglucinol derivatives known as bitter acids. Origin of the extract was guaranteed by producer, and qualitative standard was defined as bitter acid content resulting as 4.7 and 6.1% in α-acids and β-acids, respectively. The extract was further investigated for the phenolic characterization, and total phenolic, flavonoid, tannin, and carotenoid profile was determined through validated colorimetric tests. The experimental procedures for all assays were comprehensively described in our previous papers (Orlando et al., 2019). Hop extract (5 μg/mL) was analyzed by an independent and quantitative determination of phenol fraction using a reversed-phase high-performance liquid chromatography (HPLC)–fluorimetric method in gradient elution mode. Analyses were carried out by using a liquid chromatograph (MOD. 1525; Waters Corporation, Milford, MA, United States) equipped with a fluorimetric detector (MOD. 2475; Waters Corporation), a C18 reversed-phase column (Acclaim TM 120, 3 μm, 2.1 × 100 mm; Dionex Corporation, Sunnyvale, CA, United States), an online degasser (Biotech 4-CH DEGASi compact; Lab Service, Anzola dell’Emilia, Italy). The gradient elution was achieved by a mobile phase methanol–acetic acid–water (10:2:88, vol/vol) as solvent A and methanol–acetic acid–water (10:2:88, vol/vol) as solvent B. For analyte detection, λex = 278 nm and λem = 360 nm were selected in order to quantify the following phenolic compounds: gallic acid, catechin, epicatechin, and resveratrol. For rutin determination, λex = 340 nm and λem = 420 nm were selected.

### Biocompatibility Tests

*Artemia salina* L. cysts were hatched in oxygenated artificial sea water (1 g cysts/L). After 24 h, brine shrimp larvae were gently transferred with a pipette in a six-well plate containing 2 mL of hop extract at different concentrations (0.1–20 mg/mL) in artificial sea water. Ten larvae per well were incubated at 25 to 28°C for 24 h. After 24 h, the number of living napulii was counted under light microscope and compared to control untreated group. Results were expressed as percentage of mortality calculated as follows: [(*T* − *S*)/*T*] × 100. *T* is the total number of incubated larvae, and *S* is the number of survival napulii. Living napulii were considered those exhibiting light activating movements during 10 s of observation. For each experimental condition, three experiments were performed using two replicates.

Murine cardiomyocyte C2C12 and human colon cancer–derived HCT116 cell lines were cultured in Dulbecco modified eagle medium (Euroclone) supplemented with 10% (vol/vol) heat-inactivated fetal bovine serum and 1.2% (vol/vol) penicillin G/streptomycin in 75 cm^2^ tissue culture flask (*n* = 5 individual culture flasks for each condition). The cultured cells were maintained in humidified incubator with 5% CO_2_ at 37°C. For cell differentiation, C2C12 and HCT116 cell suspensions at a density of 1 × 10^6^ cells/mL were treated with various concentrations (10, 50, and 100 ng/mL) of phorbol myristate acetate (PMA, Fluka) for 24 or 48 h (induction phase). Thereafter, the PMA-treated cells were washed twice with ice-cold pH 7.4 phosphate-buffered solution (PBS) to remove PMA and non-adherent cells, whereas the adherent cells were further maintained for 48 h (recovery phase).

Morphology of cells was examined under an inverted phase-contrast microscope (Leica DMi1). To assess the basal effect of hop extract, a viability test was performed on 96-microwell plates, using 3-(4,5-dimethylthiazol-2-yl)-2,5-diphenyltetrazolium bromide (MTT) test. Cells were incubated with extract (ranging in the concentration 10–1,000 μg/mL) for 24 h. Ten microliters of MTT (5 mg/mL) was added to each well and incubated for 3 h. The formazan dye formed was extracted with dimethyl sulfoxide, and absorbance was recorded as previously described (Ferrante et al., 2019a). Effects on cell viability were evaluated in comparison to untreated control group.

Finally, we tested the hop extract on both cell lines, using a wound healing experimental paradigm. Cell migration was determined using the scratch wound healing assay, as previously reported (Ferrante et al., 2019b). HCT116 cells (6 × 10^3^ cells/well) were seeded on six-well plastic plates. Cell monolayers were preliminarily treated with a proliferation inhibitor mitomycin C (Sigma-Aldrich) at the non-toxic concentration of 5 μM, in order to exclude the effect of cell proliferation. After 2 h on cells in the confluence interval 85 to 90%, a wound was generated by scratching the cell monolayer using a 0 to 200 μL pipette tip. Two gentle washes with PBS were performed to remove suspended and damaged cells. Cells were incubated in serum free media supplemented with hop extract in the concentration range 10 to 1,000 μg/mL. Cell migration was followed capturing at least three microscope images per well at different time points: 0, 24, and 48 h. An inverted light Leica microscope equipped with Nikon 5100 camera was used to capture image at 4 × /10 × magnification. The quantification of scratch area with no cells was quantified using ImageJ software (National Institutes of Health). Using GraphPad software (version 5.01 for Windows; GraphPad Software, San Diego, CA, United States), mean data at T0, 24, and 48 h were calculated for untreated control and hop group and expressed as percentage variation with reference to relative 100% at 0 h.

### Bacterial Strains

The strains used for this study are listed and characterized for their susceptibility profiles in Table 1.

**TABLE 1:** Strains used in the present study.

Strains	Antimicrobial resistance pattern	References
*S. aureus* Sa1	AMC, AMP, CIP, FOX, OXA, TET	Bessa et al., 2018
*S. aureus* Sa3	AMC, AMP, CIP, FOX, IPM, OXA	Bessa et al., 2018
*S. aureus* SA007	CIP, CLI, ERI, FOX, GEN, LEV, MOX, OXA	Bessa et al., 2019
*S. aureus* SA010	CIP, CLI, DAP, ERI, FOX, LEV, MOX, OXA, TET	This study
*S. epidermidis* SE009	CIP, CLI, ERI, FOX, GEN, LEV, MOX, OXA, SXT	This study
*S. epidermidis* 317	AMP, CIP, FOX, GEN, NET, P, TET, VA	Nostro et al., 2012
*S. aureus* ATCC 29213		American Type Culture Collection
*S. epidermidis* ATCC 12228		American Type Culture Collection
*C. acnes* ATCC 11827		American Type Culture Collection

*AMC, amoxicillin/clavulanic acid; AMP, ampicillin; CLI; clindamycin; CIP, ciprofloxacin; ERI, erythromycin; FOX, cefoxitin; GEN, gentamicin; IPM, imipenem; LEV, levofloxacin; MOX, moxifloxacin; OXA, oxacillin; TET, tetracycline; SXT, trimethoprim sulfamethoxazole; NET, netilmicin; P, penicillin G; VA, vancomycin.*

The isolates *S. aureus* Sa1, *S. aureus* Sa3, *S. aureus* SA007, *S. aureus* SA010, and *S. epidermidis* SE009 were methicillin-resistant, and *S. epidermidis* 317 was an MDR strain.

*Staphylococcus epidermidis* 317 was collected from the private collection of Pharmacy Department, “G. d’Annunzio” University of Chieti, Italy, and the other clinical isolates came from the private collection at LAQV/REQUIMTE, Department of Chemistry and Biochemistry, University of Porto, Portugal.

Furthermore, *S. aureus* ATCC 29213, *S. epidermidis* ATCC 12228, and *C. acnes* ATCC 11827 were included as control strains.

The strains were stored at −80°C until use.

### Antimicrobial Action of Hop

The antimicrobial action of the hop extract was evaluated by the disk diffusion method and by the microdilution method, which allows the determination of the minimal inhibitory concentration (MIC). For the tests, *S. aureus* and *S. epidermidis* strains were grown on Mueller–Hinton (MH) agar (Liofilchem s.r.l., Roseto degli Abruzzi, Italy) under aerobic conditions at 37°C overnight, and the bacteria were suspended in cation-adjusted Mueller–Hinton broth (CAMHB, Oxoid, Madrid, Spain) in order to achieve an optical density at 600 nm (OD_600_) of 0.10. *Cutibacterium acnes* ATCC 11827 was grown in brain–heart infusion broth (BHI, Oxoid Milan, Italy) under anaerobic conditions at 37°C for 4 to 7 days, and the turbidity of the bacterial suspension was adjusted to OD_600_ of 0.14 (Weber et al., 2019). For the disk diffusion method, the above prepared suspensions were streaked evenly throughout the entire surface of an MH agar plate. Then, 6-mm filter paper disks loaded with 15 μL of hop extract at 5 mg/mL were applied to the inoculated MH agar and incubated under aerobic conditions for 24 h for staphylococci and under anaerobic conditions for 4 to 7 days for *C. acnes*. The diameter of the zone of growth inhibition around the disks was measured in millimeters. Five-micrograms of ciprofloxacin (Sigma Aldrich, Milan, Italy) was used as positive control for all tested strains.

For the MIC determination, the microdilution method was used. Briefly, the standardized bacterial suspensions (described above) were diluted 1:100 in MH broth (staphylococci) or in BHI (*C. acnes*) and used to inoculate 96-well microtiter plates previously containing the hop extract serial diluted (2-fold dilutions) in the respective media. Thus, the final bacterial inoculum was of approximately 5 × 10^5^ cfu/mL, and the hop extract was tested in the final concentration range of 1,024 to 0.12 μg/mL. *Staphylococcus aureus* and *S. epidermidis* were incubated overnight in aerobic condition and *C. acnes* in anaerobic conditions at 37°C for 4 to 5 days (Clinical and Laboratory Standards Institute [CLSI], 2018; Marini et al., 2018). Ciprofloxacin was used as positive control for all studied strains. As negative control, only media (without the strains) was added to the different concentrations of hop extract. The lowest concentration of hop extract required to inhibit bacterial growth was defined as MIC.

Each determination was performed in three independent experiments, each in duplicate.

### Docking Calculations

Based on HPLC analysis, gallic acid, resveratrol, and rutin were selected for docking calculations, in order to evaluate their affinity toward PBP2a and β-ketoacyl-ACP synthase III (KAS III). The routine steps for docking calculations involved the preparation of the inhibitors and the protein. The crystal structures of the PBP2a and KAS III were downloaded from Protein Data Bank (PDB). The PDB codes of PBP2a and KAS III were 6A9N and 4CJN, respectively. In order to prepare the protein for docking calculations, all water molecules and cocrystallized compounds were removed. This step was followed by adding polar hydrogen atoms and neutralized using Autodock 4 program (Molinspiration Database). The starting structures of the selected phytochemicals were optimized to their ground state structures using the AM1 semiempirical method, and the three-dimensional (3D) structures were saved in mol2 format. The protein was immersed in a 3D grid box with 60 × 60 × 60 dimensions with 0.375 Å distance between points. Lamarckian genetic algorithm was used to calculate the docking free energy of 250 conformations for each inhibitor. The docking results were clustered and organized according to the docking free energy. The binding site was localized, and the nonbonding non-bonding interactions were elucidated using Discovery Studio 5.0 visualizer.

### Membrane Fluidity Assessment by Laurdan Generalized Polarization (GP_exc_)

Eventual changes in the membrane fluidity of *S. aureus* ATCC 29213, *S. epidermidis* ATCC 12228, and *C. acnes* ATCC 11827 derived to the hop extract treatment were evaluated by assessing the Laurdan generalized polarization (GP_exc_) as previously described by Bessa et al. (2019). Briefly, fresh colonies were inoculated in nutrient broth (NB, Liofilchem s.r.l., Roseto degli Abruzzi, Italy) to obtain an OD_600_ of 0.4. Aliquots of 1.5 mL of these bacterial suspensions were taken and centrifuged (9,000 rpm, 8 min); pellets were resuspended in 1.5 mL of NB (in duplicate, to serve as controls; one to be unlabeled and the other to be labeled with Laurdan) and NB containing 2 MIC, MIC, and 1/2 MIC of hop extract and incubated at 37°C for 3 h. After incubation, the bacterial suspensions were centrifuged (9,000 rpm, 8 min), and cells were washed twice with 15 mM Tris–HCl buffer (pH 7.4) and finally resuspended in 10 μM of Laurdan (from a 2 mM stock solution in dimethylformamide) and incubated in the dark at 37°C with shaking (500 rpm) for 1.5 h. Laurdan emission spectra were obtained in a Varian Cary Eclipse fluorescence spectrofluorometer (Agilent Technologies, Santa Clara, CA, United States) at an excitation wavelength of 350 nm using emission wavelengths from 410 to 550 nm. The excitation GP_exc_ was calculated using the following equation: GP_exc_ = (I440 − I490)/(I440 + I490), where I440 and I490 are fluorescence intensities at 440 and 490 nm, respectively.

As positive control, the GP_exc_ of ciprofloxacin at 2 MIC, MIC, and 1/2 MIC concentrations was determined as describe above.

### Antibiofilm Action of Hop

The effect of hop extract on the biofilm formation by *S. aureus*, *S. epidermidis*, and *C. acnes* strains was assessed. *Staphylococcus aureus* and *S. epidermidis* strains were grown in MH agar under aerobic conditions at 37°C overnight, and the fresh colonies were used to prepare the bacterial inoculum in trypticase soy broth (TSB, Liofilchem s.r.l., Roseto degli Abruzzi, Italy) with an OD_600_ of 0.10. *Cutibacterium acnes* ATCC 11827 was grown in BHI (Oxoid, Milan, Italy) under anaerobic conditions at 37°C for 4 to 7 days and the turbidity of the bacterial suspension was adjusted to OD_600_ of 0.14 in BHI +1% glucose (Weber et al., 2019).

The adjusted inoculum was further diluted (1:100) in each broth and inoculated in 96-well flat-bottom microtiter plates in presence of MIC, 1/2 MIC, 1/4 MIC, or without (control) hop extract. The plates were then incubated at 37°C for 24 h in aerobic condition for staphylococci and for 3 days in anaerobic condition for *C. acnes*, allowing the biofilm formation. As negative control, only media (without the strains) was added to the different concentrations of hop extract. After incubation, the planktonic phase was removed; biofilms were washed with sterile PBS, air-dried, stained for 5 min with 0.5% crystal violet, washed with distillated water, and air-dried. The stained and dry biofilms were resuspended in 200 μL acetic acid (33% vol/vol), and the OD_595_ was measured. Each condition was tested in three independent experiments, each in triplicate (three wells).

Ciprofloxacin, at MIC values, was used as positive control for all detected strains (Marini et al., 2019).

Additionally, a qualitative evaluation to examine cell viability within biofilms formed in presence of MIC, 1/2 MIC, and 1/4 MIC of hop extract was also performed through the live/dead staining kit (Molecular Probes Inc., Invitrogen, San Giuliano Milanese, Italy) as indicated by the manufacturer and visualized under a fluorescence Leica 4000 DM microscope. The biofilms were formed as mentioned above in μ-Dish (35 mm, high); ibidi Polymer Coverslips (ibidi GmbH, Planegg-Martinsried, Germany) were used (2 mL/dish). Prior to microscopic observation, planktonic phases were removed; biofilms were washed with PBS and then stained with the live/dead staining mixture for 15 min in the dark, washed once again, and then examined under the fluorescence microscope (Di Lodovico et al., 2019).

### Statistical Analysis

Statistical analysis was performed using GraphPad Prism version 5.01 for Windows. Data were obtained from at least three independent experiments performed in duplicate. Data were shown as the means ± standard deviation (SD). The results regarding the biofilm formation were expressed as mean values ± SD. Differences between control and hop extract treated groups were assessed with paired Student *t* test. *P* ≤ 0.05 was considered statistically significant.

## Results

### Phytochemical Profile

As shown in Table 2, colorimetric assays showed the presence of multiple classes of phenolic compounds, namely, phenolic acids, flavonoids, tannins, and carotenoids, whose presence was confirmed by independent HPLC-fluorimetric analysis. The HPLC analysis permitted to measure the levels of gallic acid, resveratrol, and rutin, in the hop extract. In this regard, rutin level was higher than the other assayed compounds.

**TABLE 2:** Phytochemical composition of hop extract.

Total phenols (as mg of gallic acid equivalents per mL of extract)	0.24 ± 0.08 mg/mL
Total flavonoids (as mg of rutin equivalents per mL of extract)	0.07 ± 0.01 mg/mL
Total tannins (as mg of tannic acid equivalents per mL of extract)	0.017 ± 0.03 mg/mL
Total carotenoid pigments (mg/mL)	0.44 ± 0.08 mg/mL
Gallic acid	0.038 ± 0.01 μg/mL
Catechin	Not detected
Epicatechin	Not detected
Resveratrol	<0.001 μg/mL
Rutin	0.63 ± 0.04 μg/mL

### Biocompatibility Tests

**FIGURE 1:**
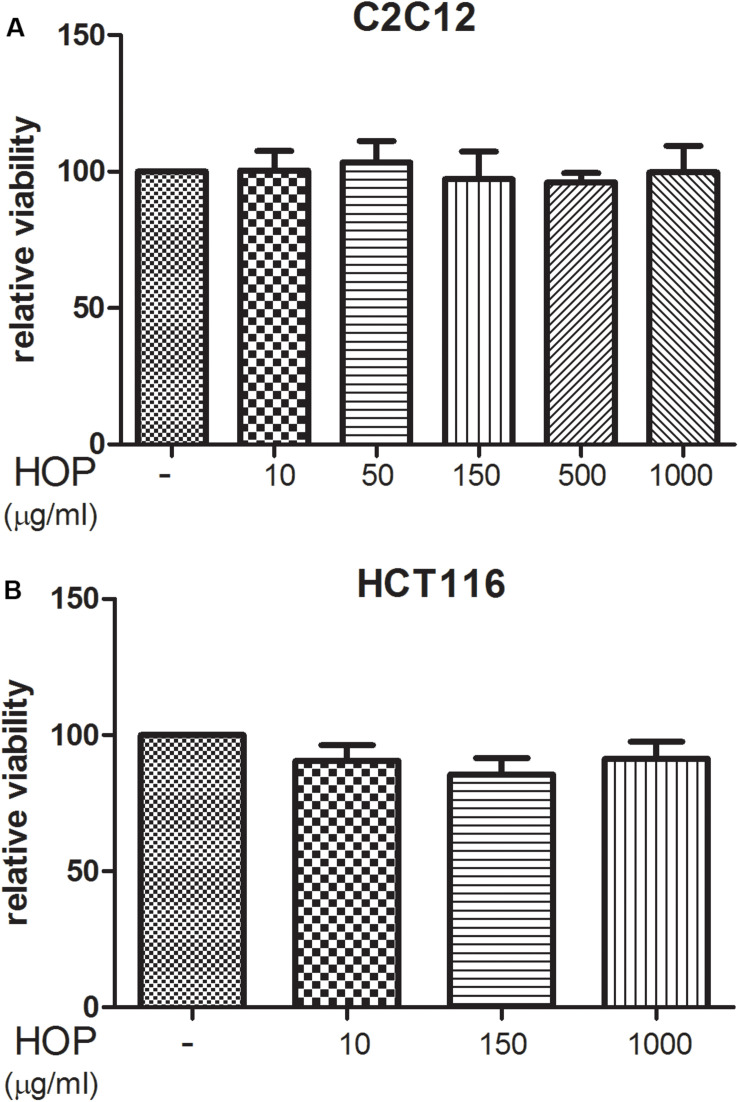
Hop extract effects on cell viability (24 h) on **(A)** murine cardiomyocyte C2C12 and **(B)** human colon cancer–derived HCT116 cell lines.

**FIGURE 2:**
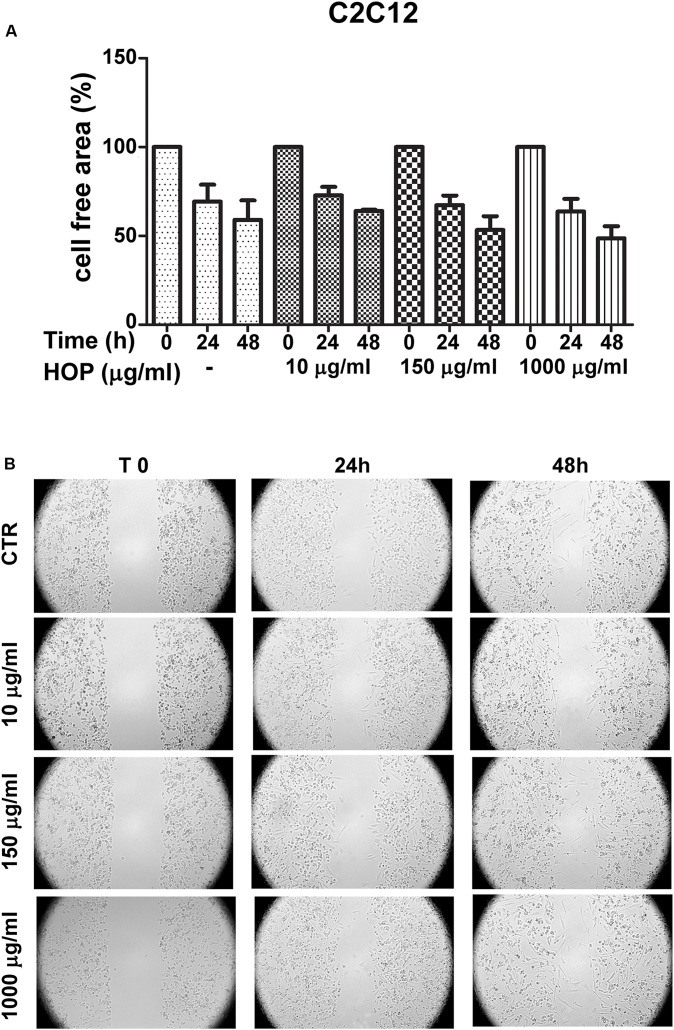
Effects of subtoxic concentration of hop extract on spontaneous cell migration in murine cardiomyocyte C2C12. Quantification of free cell area **(A)** and representative images **(B)** of wound healing test (10–1,000 μg/mL) recorded at T 0, 24, and 48 h (4 × magnification).

**FIGURE 3:**
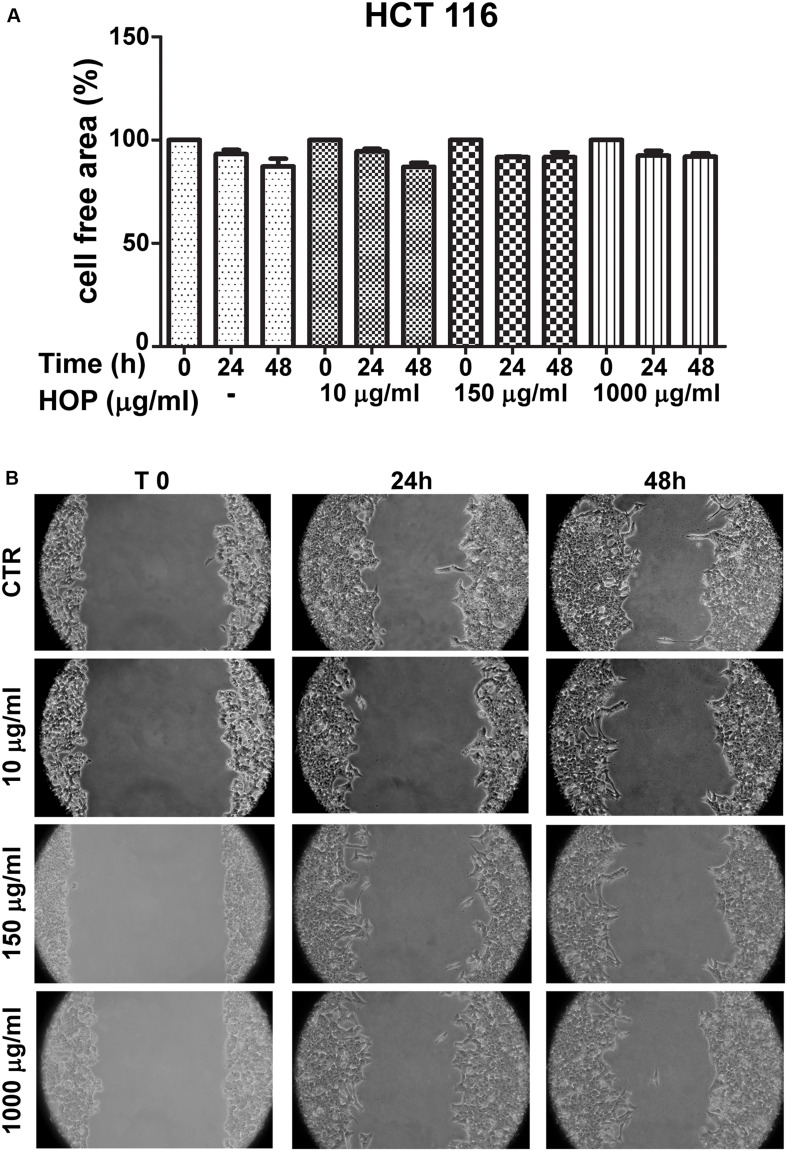
Effects of subtoxic concentration of hop extract on spontaneous cell migration in human colon cancer–derived HCT116 cell line. Quantification of free cell area **(A)** and representative images **(B)** of wound healing test (10–1,000 μg/mL) recorded at T 0, 24, and 48 h (10 × magnification).

To explore the biocompatibility, the hop extract (1–20 mg/mL) was tested in the *A. salina* L., using a lethality assay on brine shrimps. Because the results of the test indicated an LC_50_ value > 10 mg/mL, a concentration range at least 10-fold lower was then chosen for the following *in vitro* tests. Therefore, the hop extract was tested in the non-tumoral C2C12 and tumoral HCT116 cells, in the concentration range 10 to 1,000 μg/mL (Figure 1). Particularly, the extract was biocompatible at all tested concentrations, with cell viability ranging from 80 to 100%, compared to control group. Furthermore, the wound healing test ruled out any involvement in spontaneous migration of C2C12 and HCT116 cells, after challenging with the hop extract (Figures 2, 3).

### Antimicrobial Action of Hop

The hop extract exhibited antibacterial activity against all strains used in this study, including MDR clinical isolates (Table 3). The diameter of the zones of inhibition caused by hop extract was relatively variable among the strains tested (ranged between 12 and 21 mm).

**TABLE 3:** Zones of inhibition (mm) and minimum inhibitory concentration (MIC) values (μg/mL) of hop extract and ciprofloxacin (CIP) against *Staphylococcus aureus*, *Staphylococcus epidermidis*, and *Cutibacterium acnes* strains.

Strains	Hop extract		CIP	
	Zone of inhibition	MIC	Zone of inhibition	MIC
*S. aureus* ATCC 29213	12	16	25	0.25
*S. aureus* Sa1	20	16	0	128
*S. aureus* Sa3	12	16	0	128
*S. aureus* SA007	19	16	0	64
*S. aureus* SA010	12	8	0	64
*S. epidermidis* ATCC 12228	16	16	21	0.12
*S. epidermidis* SE009	18	16	0	32
*S. epidermidis* 317	17	8	15	2
*C. acnes* ATCC 11827	21	1	19	1

The MIC values of hop extract ranged from 1 to 16 μg/mL (Table 3). The lower MIC value was obtained against *C. acnes* ATCC 11827 (1 μg/mL). Moreover, there was no apparent correlation between zones of inhibition and MIC values; higher zone of inhibition did not always correspond to lower MIC values, as, for instance, comparing the results obtained for *S. aureus* ATCC 29213 and *S. aureus* Sa1.

The ciprofloxacin zones of inhibition ranged from 0 to 25 mm. All clinical isolates were resistant to ciprofloxacin, as expected (Table 1).

The ciprofloxacin MICs ranged from 0.12 to 128 μg/mL.

### Docking Analysis

**FIGURE 4:**
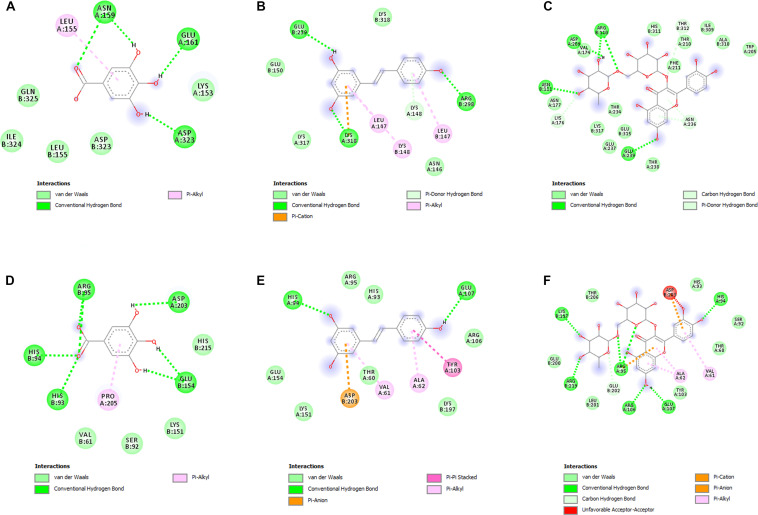
**(A–F)** Interactions of the docked compounds, gallic acid, resveratrol, and rutin. The 2D orientations of the docked compounds are shown. The putative interactions with PBP2a (PDB: 4CJN) and KAS III (PDB: 6A9N) are shown with different colors. **(A)** Gallic acid vs. PBP2a (PDB:4CJN): ΔG = –5.5 kcal/mol; Ki = 94.10 μM; **(B)** resveratrol vs. PBP2a (PDB:4CJN): ΔG = –6.7 kcal/mol; Ki = 12.40 μM; **(C)** Rutin vs. PBP2a (PDB:4CJN): ΔG = –8.5 kcal/mol; Ki = 0.60 μM; **(D)** gallic acid vs. KAS III (PDB:6A9N): ΔG = –5.7 kcal/mol; Ki = 67.18 μM; **(E)** resveratrol vs. KAS III (PDB:6A9N): ΔG = –6.8 kcal/mol; Ki = 10.52 μM; **(F)** rutin vs. KAS III (PDB:6A9N): ΔG = –9.0 kcal/mol; Ki = 0.26 μM.

In order to deeply investigate the molecular interactions underlying the observed antimicrobial effects, gallic acid, resveratrol, and rutin were docked against PBP2a and KAS III. The docking approach permitted to elucidate the orientation of the selected phenols against these microbial proteins, at their respective active sites. The related binding affinities, measured as binding free energies (ΔG), the inhibition constant (Ki), and the non-bonding interactions, were evaluated as well and reported in Figures 4A–F. Among tested compounds, rutin revealed to be the most potent against both PBP2a and KAS III, with Ki values in the submicromolar range.

### Membrane Fluidity Assessment by Laurdan GP_exc_

To evaluate if hop extract could affect the bacterial membrane, specifically, the membrane fluidity, Laurdan GP_exc_ values were calculated. Higher Laurdan GP_exc_ values correspond to lower membrane fluidity. Laurdan GP_exc_ values increased with increasing concentrations of hop extract for all tested strains, as shown in Table 4. Therefore, hop extract tended to decrease the membrane fluidity. The GP_exc_ values obtained with ciprofloxacin were similar to controls for all tested strains.

**TABLE 4:** GP_exc_ values of *Staphylococcus aureus* ATCC 29213, *Staphylococcus epidermidis* ATCC 12228, and *Cutibacterium acnes* ATCC 11827 treated with 2 MIC, MIC, and 1/2 MIC of hop extract and ciprofloxacin (CIP) compared with the untreated controls.

Strains		Hop extract			CIP		
	Control	2 MIC	MIC	1/2 MIC	2 MIC	MIC	1/2 MIC
*S. aureus* ATCC 29213	0.236	0.290	0.280	0.252	0.242	0.243	0.245
*S. epidermidis* ATCC 12228	0.197	0.203	0.193	0.196	0.186	0.189	0.161
*C. acnes* ATCC 11827	0.251	0.311	0.308	0.277	0.257	0.258	0.256

### Antibiofilm Action of Hop

Hop extract displayed a remarkable inhibition of biofilm formation at sub-MIC values for all tested microorganisms. A significant biofilm biomass reduction was recorded for all tested strain (except for *S. aureus* ATCC 29213 and the clinical isolate *S. aureus* SA010) in presence of sub-MIC values of hop extract in respect to the controls (Table 5).

**TABLE 5:** Inhibition of biofilm formation (%) by *Staphylococcus aureus*, *Staphylococcus epidermidis*, and *Cutibacterium acnes* strains in presence of MICs and sub-MICs of hop extract compared to the ciprofloxacin (CIP) in respect to the control samples (absence of hop extract).

% of biofilm reduction				
	Hop extract			CIP
Strains	MIC	1/2 MIC	1/4 MIC	MIC
*S. aureus* ATCC 29213	95.33* ± 2.20	0.00 ± 0.00	0.00 ± 0.00	92.83* ± 1.55
*S. aureus* Sa1	97.49* ± 1.84	93.88* ± 5.64	34.60* ± 3.68	95.97* ± 0.63
*S. aureus* Sa3	95.60* ± 3.15	83.53* ± 2.29	34.12* ± 1.37	87.08* ± 3.60
*S. aureus* SA007	95.57* ± 2.72	96.76* ± 1.70	35.29* ± 2.69	99.41* ± 0.12
*S. aureus* SA010	96.45* ± 4.55	0.00 ± 0.00	0.00 ± 0.00	100* ± 0.00
*S. epidermidis* ATCC 12228	86.96* ± 3.18	44.20* ± 1.66	43.08* ± 10.36	89.25* ± 2.30
*S. epidermidis* SE009	98.29* ± 2.41	95.04* ± 2.05	31.63* ± 2.54	86.86* ± 2.20
*S. epidermidis* 317	99.34* ± 0.26	98.74* ± 1.64	62.13* ± 0.83	95.43* ± 4.80
*C. acnes* ATCC 11827	84.18* ± 2.65	33.36* ± 3.84	30.97* ± 1.44	95.09* ± 4.30

*Statistically significant in respect to the control (*P* < 0.05).

At sub-MIC values, the best antibiofilm effect was detected at 1/2 MIC against *S. epidermidis* 317 with 98.74 ± 1.64% of biomass reduction in respect to the control (absence of hop extract). Interestingly, at 1/4 MIC, the biomass reductions were significant in respect to the controls and ranged from 62.13 ± 0.83% against *S. epidermidis* 317 and 30.97 ± 1.44% against *C. acnes* ATCC 11827. The obtained percentages of biofilm inhibition in presence of hop extract were similar to those detected with ciprofloxacin.

**FIGURE 5:**
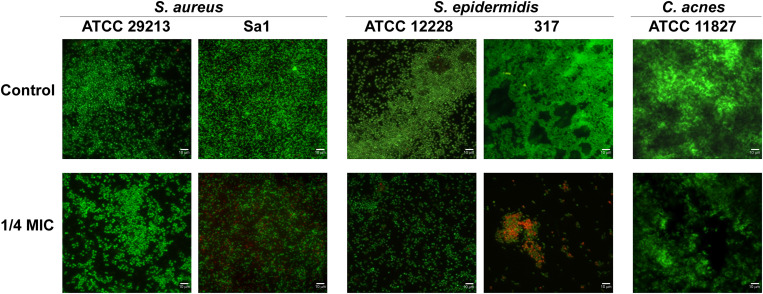
Representative live/dead images of *S. aureus* ATCC 29213, *S. aureus* Sa1, *S. epidermidis* ATCC 12228, *S. epidermidis* 317, and *C. acnes* ATCC 11827 biofilms in presence of 1/4 MIC of hop extract compared to the respective controls. The images observed by fluorescent Leica 4000 DM microscopy (Leica Microsystems, Milan, Italy) were recorded at an emission wavelength of 500 nm for SYTO 9 and of 635 nm for propidium iodide, and several fields of view were randomly examined. Original magnification × 1,000.

The effect of hop extract in inhibiting the biofilm formation at sub-MIC values was also observed through live/dead staining for all detected strains. Representative images are shown in Figure 5. Except for *S. aureus* ATCC 29213, the biofilms formed by staphylococci in presence of hop extract were clearly less abundant in respect to the controls, presenting also a weak reduction in the viability. In fact, a moderate killing action was detected up to 1/4 MIC values for the clinical strains of *S. aureus* and *S. epidermidis.* A weak reduction of cell adhesion with no significant differences in terms of viability, however, was observed for the three reference strains assayed when comparing biofilms formed in presence of 1/4 MIC of hop extract and in absence of it (Figure 5).

## Discussion

In this study, the antimicrobial and antibiofilm properties of hop extract were evaluated against staphylococci strains and *C. acnes* including multiresistant isolates. The global increase in multidrug resistance underlines the need to search for new strategies to control the microorganism proliferation and to halt their associated infections.

Nowadays, the discovery of new antibiotics is a lengthy and expensive process, and the bacterial capability to develop resistance is unpredictable and fast (Brown, 2015). For these reasons, natural extracts have been proposed as alternative or adjuvant antimicrobial agents. In this regard, hop extract certainly deserves to be further explored. Previous studies showed a significant antibacterial action of hop extract against different Gram-positive pathogenic bacteria (Weber et al., 2019).

The main hop components are bitter acids, usually classified as α- and β-acids and the derived isomers such as *cis*-iso-α-acids and *trans*-iso-α-acids. Among them, the iso-α-acids could be considered the most relevant for their bitter organoleptic characters and for their antibacterial properties (Teuber and Schmalreck, 1973).

The plant used in this study is a variety selected for specific flavoring effects related to the balance between α- and β-acids, useful in beer production. The phenolic fraction revealed the presence of a complex mixture of a different class of secondary metabolites that, if present in low amount, can significantly influence the organoleptic and biological effects, including the antibacterial and antibiofilm activities. Among the tested phenolic compounds, rutin was present at higher concentrations compared to the other phytochemicals. This is consistent, albeit partially, in recent literature data (Almeida et al., 2020).

The hop extract was biocompatible at all tested concentrations showing significant viable cells in both tested murine cardiomyocyte C2C12 and human colon cancer–derived HCT116 cell lines.

Overall, the *in vitro* tests, besides confirming the biocompatibility of the extract, any cytotoxicity that often occurs with herbal extracts were excluded, especially at high concentrations (1,000 μg/mL) (Orlando et al., 2020; Recinella et al., 2020). The wound healing test also ruled out any influence of this extract in modifying the intrinsic spontaneous migration of both tested cell lines.

Moreover, the hop extract displayed a very good antimicrobial action against the strains included in this study with MIC values widely lower than those used to demonstrate hop biocompatibility. The results related to the zones of growth inhibition and MIC values seem apparently discordant between them. This may be related to the wide range of bioactive molecules composing the hop extract, as well as to its probable hydrophobic nature, which prevents the uniform diffusion and solubility of these active components through the agar medium (Hammer et al., 1999). Therefore, the disk diffusion method may not be the best to assess the antibacterial effect of hop extract. MIC values may mirror better the antibacterial activity of hop extract on the tested strains.

The antibacterial activity of hop extract, especially its good effect against MDR Gram-positive isolates, may be attributed to the phenolic compounds as also reported by Bocquet et al. (2019), who concluded that the phenolic compounds had more effect against staphylococci and in particular against MRSA strains. Probably, the phenolic compounds are able to break the antibiotic resistance. Regarding the action mechanism of hop extract, Rozalski et al. (2013) reported that hop compounds affected the cell wall and the membrane integrity and could also bind the PBP inside the wall. Rutin, the main component present in our tested hop extract, showed the highest affinity toward PBP2a, thus further highlighting the importance of this flavonoid in the antimicrobial effects exerted by hop and other herbal extracts (Orlando et al., 2020). Interestingly, hop extract showed a strong antibacterial action against the anaerobic *C. acnes* with very low MIC values (1 μg/mL) likely due to its capability to inhibit the *C. acnes* lipase (Falcocchio et al., 2006). β- and α-acids produce an intracellular accumulation of protons, dissipation of the transmembrane proton gradient, decrease in proton motive force (pmf)-driven uptake of nutrients, and starvation and cause cell growth inhibition (Karabın et al., 2016). Moreover, rutin also showed a high affinity to KAS III, determining the inhibition of fatty acid synthesis, necessary for the survival of *C. acnes* (Cheon et al., 2019).

Gerhäuser (2005) showed that bitter acid, extracted from the hop, could interact with the bacterial cell wall and consequently exert its antibiotic action. Here, we aimed to evaluate if hop extract could affect the bacterial membrane by assessing changes in membrane fluidity through the calculation of Laurdan GP_exc_ values.

Fluidity is associated with the degree of geometrical packing of the phospholipid membrane, a process governed by polar head group composition and fatty acyl chain conformation (Bessa et al., 2018). The slight rigidity (increase in Laurdan GP_exc_ values) of the membrane caused by hop extract in all three tested strains may allow anticipating the effect of hop extract on the cytoplasmic bacterial membrane. This is in accordance with previous reports that have attributed the antibacterial effect of hop extract to the interference of prenyl groups (hydrophobic groups) of some hop acids and flavonoids with the cytoplasmic membrane (Bartmańska et al., 2018; Bocquet et al., 2019).

In this study, it was also found that hop extract affected the biofilm formation of all tested Gram-positive bacteria. Biofilms formed in presence of subinhibitory concentrations of hop extract were significantly reduced and showed less clustered cells. Moreover, biofilms formed by *S. aureus* and *S. epidermidis* clinical isolates in presence of hop extract revealed lower viability, which can be noticed even though a quantitative assessment on the viability was not undertaken.

Hop extract may interfere with adhesion that is an important step in biofilm formation. In addition, Bogdanova et al. (2018) correlated the antibiofilm effect of hop with its capability to interfere with quorum-sensing against Gram-positive bacteria.

Overall, the hop extract showed antimicrobial and antibiofilm activities against *S. aureus*, *S. epidermidis*, and *C. acnes* strains including the MDR clinical isolates. Considering that the concentrations of hop extract at which it exerted its antibacterial action were lower than those employed in the biocompatibility tests, causing no cytotoxicity and no spontaneous migration in the tested cell lines, this hop extract may have the potential to be further explored for its use and application in the treatment and management of infections caused by multidrug Gram-positive bacteria. Future studies will be needed to confirm lack of cytotoxicity on skin epithelial cells or fibroblasts in order to validate the topical application of this hop extract.

## Data Availability Statement

The raw data supporting the conclusions of this article will be made available by the authors, without undue reservation.

## Author Contributions

All authors made significant contributions to this article and participated actively in the conception and design of the experiments, read and approved the final manuscript. SD, ER, LB, and JC-A conducted the experiments. LM and CF performed the phytochemical assays. SD, LM, PG, LC, and LB performed data analysis. SD and LC wrote the manuscript. SD, LM, CF, PG, LB, and LC contributed to discussing the results and critical review of the manuscript.

## Conflict of Interest

The authors declare that the research was conducted in the absence of any commercial or financial relationships that could be construed as a potential conflict of interest.

## References

[B1] Achermann Y. Goldstein E. J. Coenye T. Shirtliff M. E. (2014). *Propionibacterium acnes*: from commensal to opportunistic biofilm-associated implant pathogen. *Clin. Microbiol. Rev.* 27 419–440. 10.1128/CMR.00092-13 24982315 PMC4135900

[B2] Almeida A. R. de Oliveira Brisola Maciel M. V. Machado M. H. Bazzo G. C. Dutra de Armas R. Bitencourt Vittorino V. (2020). Bioactive compounds and antioxidant activities of Brazilian hop (*Humulus lupulus* L.) extracts. *Int. J. Food Sci. Tech.* 55 340–347. 10.1111/ijfs.14311

[B3] Bartmańska A. Wałecka-Zacharska E. Tronina T. Popło’nski J. Sordon S. Brzezowska E. (2018). Antimicrobial properties of spent Hops extracts, flavonoids isolated there from and their derivatives. *Molecules* 23:2059. 10.3390/molecules23082059 30126093 PMC6222488

[B4] Becker K. Heilmann C. Peters G. (2014). Coagulase-negative staphylococci. *Clin. Microbiol. Rev.* 27 870–926. 10.1128/CMR.00109-13 25278577 PMC4187637

[B5] Bessa L. J. Ferreira M. Gameiro P. (2018). Evaluation of membrane fluidity of multidrug-resistant isolates of *Escherichia coli* and *Staphylococcus aureus* in presence and absence of antibiotics. *J. Photochem. Photobiol. B* 181 150–156. 10.1016/j.jphotobiol.2018.03.002 29567316

[B6] Bessa L. J. Manickchand J. R. Eaton P. Leite J. R. S. A. Brand G. D. Gameiro P. (2019). Intragenic antimicrobial peptide Hs02 hampers the proliferation of single- and dual-species biofilms of *P. aeruginosa* and *S. aureus*: a promising agent for mitigation of Biofilm-associated infections. *Int. J. Mol. Sci.* 20:3604. 10.3390/ijms20143604 31340580 PMC6678116

[B7] Blumenthal M. Goldberg A. Brinckmann J. (2000). *Herbal Medicine: Expanded Commission Monographs.* Newton: Integrative Medicine Communications 297–303.

[B8] Bocquet L. Sahpaz S. Bonneau N. Beaufay C. Mahieux S. Samaillie J. (2019). Phenolic compounds from Humulus lupulus as natural antimicrobial products: new weapons in the fight against methicillin resistant *Staphylococcus aureus*, *Leishmania mexicana* and *Trypanosoma brucei* strains. *Molecules* 24:1024. 10.3390/molecules24061024 30875854 PMC6472001

[B9] Bogdanova K. Röderova M. Kolar M. Langova K. Dusek M. Jost P. (2018). Antibiofilm activity of bioactive hop compounds humulone, lupulone and xanthohumol toward susceptible and resistant staphylococci. *J. Res Microbiol.* 169 127–134. 10.1016/j.resmic.2017.12.005 29407045

[B10] Brown D. (2015). Antibiotic resistance breakers: can repurposed drugs fill the antibiotic discovery void? *Nat. Rev. Drug Discov.* 14 821–832. 10.1038/nrd4675 26493767

[B11] Cheon D. Kim J. Jeon D. Shin H. C. Kim Y. (2019). Target proteins of phloretin for its anti-inflammatory and antibacterial activities against *Propionibacterium acnes*-induced skin infection. *Molecules* 24:1319. 10.3390/molecules24071319 30987239 PMC6479541

[B12] Clinical and Laboratory Standards Institute [CLSI] (2018). *Methods for Dilution Antimicrobial Susceptibility Tests for Bacteria that Grow Aerobically*, 11th Edn Wayne, PA: CLSI.

[B13] Coates R. Moran J. Horsburgh M. J. (2014). Staphylococci: colonizers and pathogens of human skin. *Future Microbiol.* 9 75–91. 10.2217/fmb.13.145 24328382

[B14] Coenye T. Peeters E. Nelis H. J. (2007). Biofilm formation by *Propionibacterium acnes* is associated with increased resistance to antimicrobial agents and increased production of putative virulence factors. *Res. Microbiol.* 158 386–392. 10.1016/j.resmic.2007.02.001 17399956

[B15] Di Lodovico S. Napoli E. Di Campli E. Di Fermo P. Gentile D. Ruberto G. (2019). *Pistacia vera* L. oleoresin and levofloxacin is a synergistic combination against resistant Helicobacter pylori strains. *Sci. Rep.* 9:4646. 10.1038/s41598-019-40991-y 30874618 PMC6420558

[B16] Falcocchio S. Ruiz C. Pastor F. Saso L. Diaz P. (2006). *Propionibacterium acnes* GehA lipase, an enzyme involved in acne development, can be successfully inhibited bydefined natural substances. *J. Mol. Catal. B Enzymat.* 40 132–137. 10.1016/j.molcatb.2006.02.011

[B17] Ferrante C. Recinella L. Ronci M. Menghini L. Brunetti L. Chiavaroli A. (2019a). Multiple pharmacognostic characterization on hemp commercial cultivars: focus on inflorescence water extract activity. *Food Chem. Toxicol.* 125 452–461. 10.1016/j.fct.2019.01.035 30711720

[B18] Ferrante C. Recinella L. Ronci M. Orlando G. Di Simone S. Brunetti L. (2019b). Protective effects induced by alcoholic *Phlomis fruticosa* and *Phlomis herba*-venti extracts in isolated rat colon: focus on antioxidant, anti-inflammatory, and antimicrobial activities in vitro. *Phytother. Res.* 33 2387–2400. 10.1002/ptr.6429 31322313

[B19] Gerhäuser C. (2005). Broad spectrum antiinfective potential of xanthohumol from hop (*Humulus lupulus* L.) in comparison with activities of other hop constituents and xanthohumol metabolite. *Mol. Nutr. Food Res.* 49 827–831. 10.1002/mnfr.200500091 16092071

[B20] Grice E. A. Kong H. H. Renaud G. Young A. C. Bouffard G. G. Blakesley R. W. (2008). A diversity profile of the human skin microbiota. *Genom. Res.* 18 1043–1050. 10.1101/gr.075549.107 18502944 PMC2493393

[B21] Hammer K. A. Carson C. F. Riley T. V. (1999). Antimicrobial activity of essential oilsand other plant extracts. *J. Appl. Microbiol.* 86 985–990. 10.1046/j.1365-2672.1999.00780.x 10438227

[B22] Isard O. Knol A. C. Ariès M. F. Nguyen J. M. Khammari A. Castex-Rizzi N. (2011). *Propionibacterium acnes* activates the IGF-1/IGF-1R system in the epidermis and induces keratinocyte proliferation. *J. Invest. Dermatol.* 131 59–66. 10.1038/jid.2010.281 20927124

[B23] Karabın M. Hudcova T. Jelınek L. Dostalek P. (2016). Biologically active compoundsfrom Hops and prospects for their use. *Comprehen. Rev. Food Sci. Food Saf.* 15 542–567. 10.1111/1541-4337.12201 33401815

[B24] Lee A. S. de Lencastre H. Garau J. Kluytmans J. Malhotra-Kumar S. Peschel A. (2018). Methicillin-resistant Staphylococcus aureus. *Nat. Rev. Dis. Primers* 31:18033. 10.1038/nrdp.2018.33 29849094

[B25] Levin-Reisman I. Ronin I. Gefen O. Braniss I. Shoresh N. Balaban N. Q. (2017). Antibiotic tolerance facilitates the evolution of resistance. *Science* 355 826–830. 10.1126/science.aaj2191 28183996

[B26] Lu M. Dai T. Murray C. K. Wu M. X. (2018). Bactericidal property of oregano oil against multidrug-resistant clinical isolates. *Front. Microbiol.* 9:2329. 10.3389/fmicb.2018.02329 30344513 PMC6182053

[B27] Marini E. Di Giulio M. Ginestra G. Magi G. Di Lodovico S. Marino A. (2019). Efficacy of carvacrol against resistant rapidly growing mycobacteria in the planktonic and biofilm growth mode. *PLoS One* 14:e0219038. 10.1371/journal.pone.0219038 31260476 PMC6602199

[B28] Marini E. Di Giulio M. Magi G. Di Lodovico S. Cimarelli M. E. Brenciani A. (2018). Curcumin, an antibiotic resistance breaker against a multiresistant clinical isolate of *Mycobacterium abscessus*. *Phytother. Res.* 32 488–495. 10.1002/ptr.5994 29193368

[B29] Nostro A. Cellini L. Di Giulio M. D’Arrigo M. Marino A. Blanco A. R. (2012). Effect of alkaline pH on staphylococcal biofilm formation. *APMIS* 120 733–742. 10.1111/j.1600-0463.2012.02900.x 22882263

[B30] Oliveira D. Borges A. Simões M. (2018). *Staphylococcus aureus* toxins and their molecular activity in infectious diseases. *Toxins* 10:252. 10.3390/toxins10060252 29921792 PMC6024779

[B31] Olsovska J. Bostikova V. Dusek M. Jandovska V. Bogdanova K. Cermak P. (2016). *Humulus lupulus* L. (HOPS) – A valuable source of compounds with bioactive effects for future therapies. *Militar. Med. Sci. Lett.* 85 19–30. 10.31482/mmsl.2016.004

[B32] Orlando G. Ferrante C. Zengin G. Sinan K. I. Bene K. Diuzheva A. (2019). Qualitative chemical characterization and multidirectional biological investigation of leaves and bark extracts of *Anogeissus leiocarpus* (DC.) Guill. &Perr. (Combretaceae). *Antioxidants* 8:343. 10.3390/antiox8090343 31480498 PMC6770311

[B33] Orlando G. Recinella L. Chiavaroli A. Brunetti L. Leone S. Carradori S. (2020). Water extract from inflorescences of industrial hemp futura 75 variety as a source of anti-inflammatory, anti-proliferative and antimycotic agents: results from in silico, in vitro and ex vivo studies. *Antioxidants* 9:437. 10.3390/antiox9050437 32429587 PMC7278775

[B34] Peacock S. J. Paterson G. K. (2015). Mechanisms of Methicillin Resistance in Staphylococcus aureus. *Annu. Rev. Biochem.* 84 577–601. 10.1146/annurev-biochem-060614-034516 26034890

[B35] Platsidaki E. Dessinioti C. (2018). Recent advances in understanding *Propionibacterium acnes* (*Cutibacterium acnes*) in acne. *F1000Research* 7:F1000 Faculty Rev-1953. 10.12688/f1000research.15659.1 30613388 PMC6305227

[B36] Prescott S. L. Larcombe D. L. Logan A. C. West C. Burks W. Caraballo L. (2017). The skin microbiome: impact of modern environments on skin ecology, barrier integrity, and systemic immune programming. *World Allerg. Org. J.* 10:29. 10.1186/s40413-017-0160-5 28855974 PMC5568566

[B37] Recinella L. Chiavaroli A. Ronci M. Menghini L. Brunetti L. Leone S. (2020). Multidirectional pharma-toxicological study on *Harpagophytum procumbens* DC. ex Meisn.: an IBD-focused investigation. *Antioxidants* 9:168. 10.3390/antiox9020168 32085616 PMC7070412

[B38] Rozalski M. Micota B. Sadowska B. Stochmal A. Jedrejek D. Wieckowska-Szakiel M. (2013). Antiadherent and antibiofilm activity of *Humulus lupulus* L. derived products: new pharmacological properties. *Bio. Med. Res. Inter.* 2013:101089. 10.1155/2013/101089 24175280 PMC3794639

[B39] Sinha P. Srivastava S. Mishra N. Yadav N. P. (2014). New perspectives on antiacne plant drugs: contribution to modern therapeutics. *Bio Med. Res. Int.* 2014:301304. 10.1155/2014/301304 25147793 PMC4132408

[B40] Teuber M. Schmalreck A. F. (1973). Membrane leakage in *Bacillus subtilis*168 induced bythe hop constituents lupulone, humulone, isohumulone, and humulinic acid. *Arch. Microbiol.* 94 159–171. 10.1007/BF00416690 4205069

[B41] Weber N. Biehler K. Schwabe K. Haarhaus B. Quirin K. W. Frank U. (2019). Hop extract acts as an antioxidant with antimicrobial effects against *Propionibacterium acnes* and *Staphylococcus aureus*. *Molecules* 24:223. 10.3390/molecules24020223 30634461 PMC6359372

[B42] Williams M. R. Gallo R. L. (2015). The role of the skin microbiome in atopic dermatitis. *Curr. Allergy Asthma Rep.* 15:65. 10.1007/s11882-015-0567-4 26404536

[B43] Yamaguchi N. Satoh-Yamaguchi K. Ono M. (2009). In vitro evaluation of antibacterial, anticollagenase and antioxidant activities of hop components (*Humulus lupulus*) addressing acne vulgaris. *Phytomed. Int. J. Phytother. Phytopharmacol.* 16 369–376. 10.1016/j.phymed.2008.12.021 19201179

